# Imaging evaluation and volumetric measurement of the space surrounding the diploic veins

**DOI:** 10.1007/s11604-024-01572-w

**Published:** 2024-04-26

**Authors:** Rei Nakamichi, Toshiaki Taoka, Rintaro Ito, Tadao Yoshida, Michihiko Sone, Shinji Naganawa

**Affiliations:** 1 Department of Radiology, Nagoya University Graduate School of Medicine, 65 Tsurumai-Cho, Showa-Ku, Nagoya, 466-8550 Japan; 2 Department of Otorhinolaryngology, Nagoya University Graduate School of Medicine, 65 Tsurumai-Cho, Showa-Ku, Nagoya, 466-8550 Japan

**Keywords:** Diploic vein, Diploe, Magnetic resonance imaging, Gadolinium, Parasagittal dura

## Abstract

**Purpose:**

The diploic veins have been suggested to be involved in the excretion of cerebrospinal fluid and intracranial waste products; however, to date, there have been no reports evaluating the space surrounding the diploic veins. Therefore, we aimed to visualize the distribution of gadolinium-based contrast agent (GBCA) in the space surrounding the diploic veins and to evaluate the spatial characteristics.

**Materials and methods:**

Ninety-eight participants (aged 14–84 years) were scanned 4 h after intravenous GBCA injection at Nagoya University Hospital between April 2021 and December 2022. The volume of the space surrounding the diploic veins where the GBCA was distributed was measured using contrast-enhanced T1-weighted images with the application of three-axis motion-sensitized driven equilibrium. The parasagittal dura (PSD) volume adjacent to the superior sagittal sinus was also measured using the same images. Both volumes were corrected for intracranial volume. The correlation between age and the corrected volume was examined using Spearman’s rank correlation coefficient; the relationship between the corrected volume and sex was assessed using the Mann–Whitney *U* test.

**Results:**

A significant weak negative correlation was observed between the volume of the space surrounding the diploic veins and age (*r* = −0.330, *p* < 0.001). Furthermore, there was a significant weak positive correlation between the PSD volume and age (*r* = 0.385, *p* < 0.001). Both volumes were significantly greater in men than in women. There was no correlation between the volume of the space surrounding the diploic veins and the volume of the PSD.

**Conclusion:**

The volume of the space surrounding the diploic veins was measurable and, in contrast to the volume of the PSD, was greater in younger participants. This space may be related to intracranial excretory mechanisms and immune responses during youth, requiring further research.

## Introduction

Recently, the structural and cellular details of the skull–meninges connections near the diploic veins have been reported in humans, suggesting their involvement in immune cell migration from the skull bone marrow to the meninges [1]. Molecular translation from the cerebrospinal fluid to the diploe has also been reported, suggesting that the cranial diploe may be involved in cerebral immune surveillance [2]. The diploic veins within the cranial diploe are continuous with the superior sagittal sinus (SSS) [3]. When the SSS is occluded due to meningioma or other causes, the diploic veins may function as collateral pathways [4, 5]. The diploic veins are also reportedly continuous with the arachnoid protrusion into the skull, suggesting their involvement in the excretion of cerebrospinal fluid and intracranial waste products [6].

**Fig. 1:**
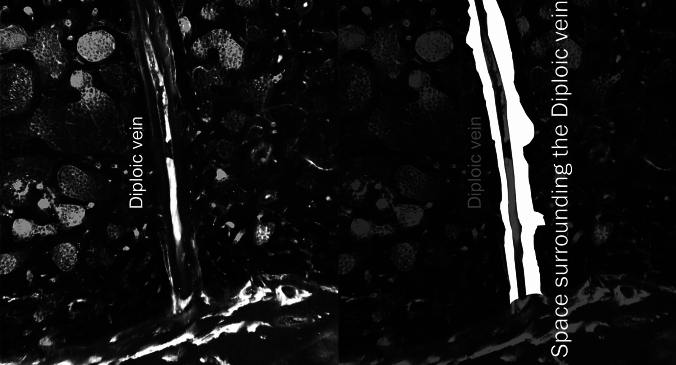
Highlighted space surrounding the lectin-labeled diploic vein in the tissue specimen of the human skull labeled with LYZ2 and lectin (reprinted and modified from a previous report [1] under Creative Common Attribution 4.0 International License)

We routinely obtain contrast-enhanced T1-weighted images, with three-axis motion-sensitized driven equilibrium (MSDE) applied, 4 h after intravenous gadolinium-based contrast agent (GBCA) injection via 3-T magnetic resonance imaging (MRI) when endolymphatic hydrops is suspected in patients with symptoms such as dizziness or tinnitus. MSDE suppresses the signal of blood flow through the vessel lumen. We noticed a high-signal region surrounding the signal-suppressed diploic veins on images with MSDE. The space surrounding the diploic veins (Fig. 1) may play a role in waste excretion and immune responses, similar to the perivascular spaces of the brain, as in the parasagittal dura (PSD) and skull–meninges connections. However, to date, no report has quantitatively evaluated the volume of this space. Therefore, this retrospective study aimed to examine the distribution and volume of the space surrounding the diploic veins where the GBCA was distributed and to analyze its spatial characteristics.

## Materials and methods

### Participants

The study included 98 (45 men and 53 women) of 99 participants who underwent an MRI examination 4 h after intravenous GBCA injection for the diagnosis of endolymphatic hydrops at Nagoya University Hospital between April 2021 and December 2022. One patient with findings indicating a brain tumor was excluded from the study. The median age of the participants was 47.25 (range, 14–84) years. None of the participants had an obvious history of brain tumors, major stroke, subarachnoid hemorrhage, head trauma, or infection of the central nervous system. The clinical research review board at Nagoya University Graduate School of Medicine approved this retrospective study (approval number: 2023–0040). Using existing anonymized information and the opt-out method, the need to obtain written informed consent was waived by the clinical research review board at Nagoya University Graduate School of Medicine.

### MRI

MSDE T1-weighted images covering the whole brain were obtained to rule out intracranial diseases 4 h after administering a single dose (0.1 mmol/kg) of gadobutrol (Gadovist; Bayer Yakuhin, Osaka, Japan), a macrocyclic GBCA. A 3-T MRI scanner (Vantage Centurian; Canon Medical Systems, Otawara, Japan) with a 32-channel head Atlas SPEEDER Head/Neck coil was used. For acquiring the three-dimensional (3D) MSDE T1-weighted transverse image on the anterior commissure–posterior commissure plane, gradients for MSDE were applied in three axes, producing an effective diffusional attenuation moment (*b* value) of 1.0 s/mm^2^. Spectral attenuated inversion recovery (SPAIR) fat suppression and deep learning reconstruction (Advanced Intelligent Clear-IQ Engine; AiCE) were used; the repetition time was 700 ms; the echo time, 15 ms; the refocus flip angle, 90°; the section increment, 0.5 mm; the voxel size, 0.3125 × 0.3125 × 1.0000 (mm); the number of slices, 320; the echo train length, 23; the field of view, 200 × 200 (mm); the matrix size, 640 × 640; the number of excitations, 1; the SPEEDER factor, ×2/×1.1; and the scan time, 4 min 54 s.

### Volumetric measurement of the space surrounding the diploic veins

**Fig. 2:**
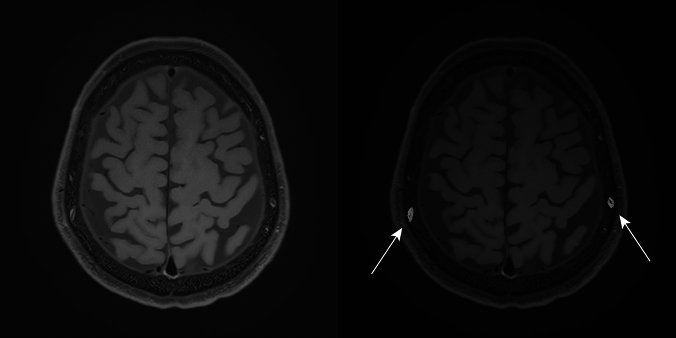
High-signal areas surrounding the diploic veins drawn as regions of interest (*arrows*) on contrast-enhanced motion-sensitized driven equilibrium (MSDE) T1-weighted transverse images

**Fig. 3:**
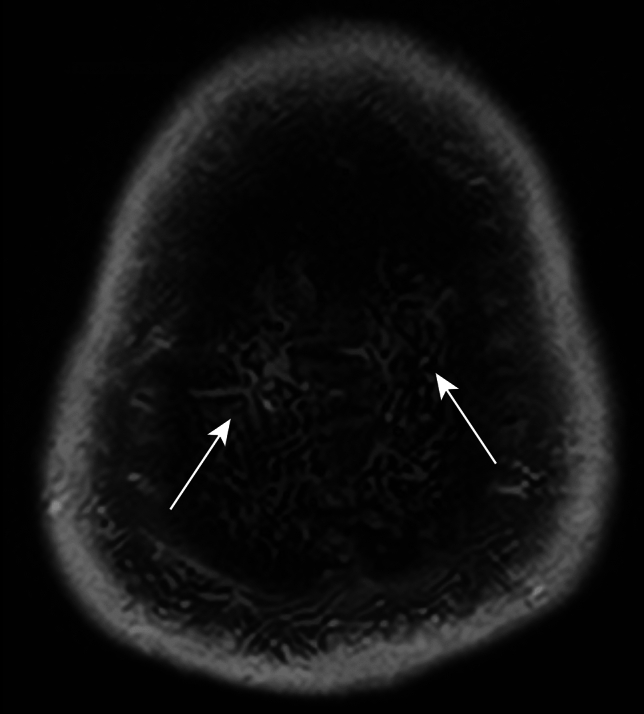
Reticular high-signal regions (*arrows*) exceeding the threshold on a contrast-enhanced motion-sensitized driven equilibrium (MSDE) T1-weighted transverse image, whose association with the diploic veins was uncertain

Transverse MSDE T1-weighted images with anonymized patient information were imported into 3D Slicer software (version 5.2.2; available at https://slicer.org). The high-signal areas surrounding the diploic veins were enclosed as regions of interest (ROIs) using a threshold-based algorithm for the slices above the superior border of the lateral ventricles (Fig. 2). The algorithm extracted regions with signal values higher than a predetermined value and then removed irrelevant structures; it was constructed with reference to previous reports [7, 8]. To avoid contrast areas other than the space surrounding the diploic veins and improve reproducibility, measurements were obtained only for high-signal areas with diameters of ≥2 mm. Additionally, reticular high-signal regions exceeding the threshold (Fig. 3) observed within the cranial diploe of certain participants, whose association with the diploic veins was uncertain, were excluded from measurement to prevent potential overestimation. The volume of the ROIs was calculated and recorded using the functions in 3D Slicer.

### Volumetric measurement of the PSD

**Fig. 4:**
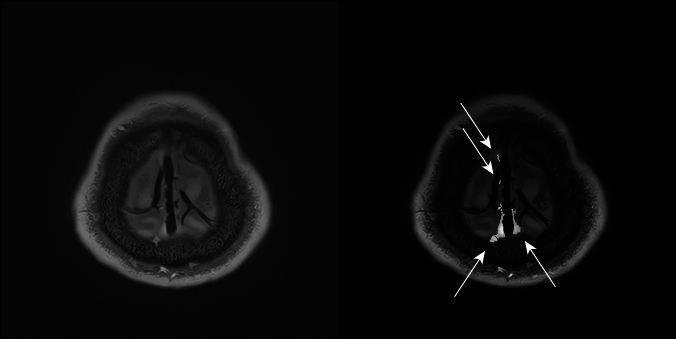
High-signal areas adjacent to the superior sagittal sinus drawn as regions of interest (*arrows*) on motion-sensitized driven equilibrium (MSDE) T1-weighted transverse images

To validate our method of measuring volume and compare the characteristics with the volume of the space surrounding the diploic veins, we measured the volume of the PSD in a similar manner. High-signal areas adjacent to the SSS were enclosed by ROIs using the same threshold-based algorithm in 3D Slicer for slices above the superior border of the lateral ventricles on the MSDE T1-weighted transverse images (Fig. 4). To avoid contrast areas other than the PSD and improve reproducibility, measurements were obtained only for high-signal areas within a 1 cm area from the outer edge of the SSS. The high-signal regions in the brain parenchyma and within the skull were manually removed from the ROIs. The volume of the ROIs was calculated and recorded using the functions in 3D Slicer.

### Assessment of the reliability of the ROIs

The ROIs were drawn by a radiologist (R.N.) with 13 years of image-reading experience. R.N. obtained measurements under conditions where clinical information was not available for reference. To assess inter-rater reliability, another radiologist (R.I.) with 10 years of image-reading experience drew the ROIs for 10 randomly selected participants using the same method as R.N. did.

### Measurement of intracranial volume

The MSDE T1-weighted transverse images were loaded into MRIcron software (version 1.0.20190902; available at https://www.nitrc.org/projects/mricron), and the brain extraction tool (fractional intensity threshold = 0.30) was applied [9]. We visually verified that no extracranial structures were included; we subsequently calculated the intracranial volume using the mask function of the volumes of interest.

### Statistical analyses

The normalities of the distributions of participants’ age, the volume of the space surrounding the diploic veins, and the volume of the PSD were evaluated using the Kolmogorov–Smirnov test. Age correlations with the volume of the space surrounding the diploic veins and the volume of the PSD were evaluated using Pearson’s product correlation coefficients for normally distributed variables and Spearman’s rank correlation coefficients for non-normally distributed variables. The relationship of sex with the volume of the space surrounding the diploic veins and the volume of the PSD was examined using the *t* test for normally distributed variables and the Mann–Whitney *U* test for non-normally distributed variables. These analyses were performed for both volumes, corrected and uncorrected for intracranial volume. We calculated the corrected volume by simply dividing the measured volume by the intracranial volume.

Inter-rater reliability for the volume of the space surrounding the diploic veins and the volume of the PSD was assessed using the intraclass correlation coefficient (ICC) (2, 1). If the volume was not normally distributed, the logarithm of the volume (Ln(*x* + 1)) was taken, and the ICC (2, 1) was calculated from the logarithm after confirming that the logarithm was normally distributed using the Kolmogorov–Smirnov test.

The ICCs were calculated using SPSS software version 28.0 (IBM, Armonk, NY, USA). Other statistical analyses were performed using EZR software version 1.61 [10]. The threshold for statistical significance was set at *p* < 0.05.

## Results

### Correlations of age and sex with the volume of the space surrounding the diploic veins and the volume of the PSD

**Fig. 5:**
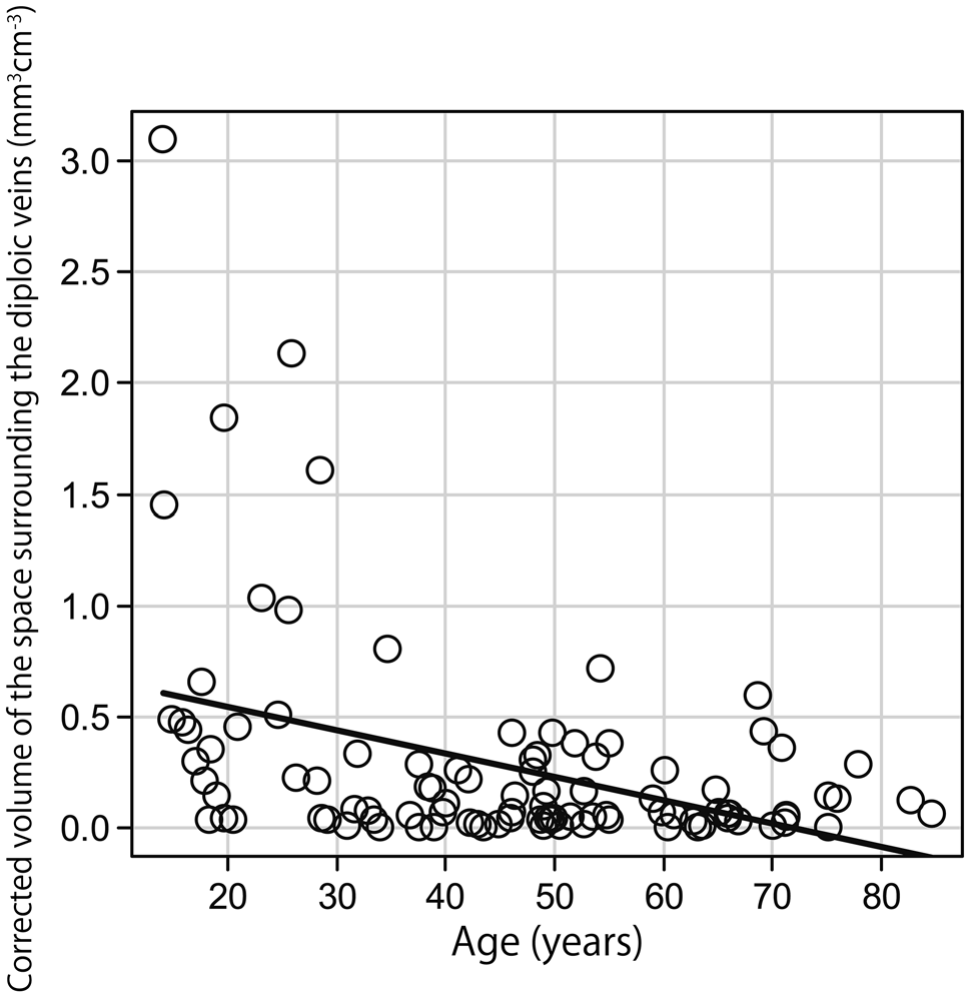
A weak but significant negative correlation (*r* = −0.330, *p* < 0.001) between age and the volume of the space surrounding the diploic veins corrected for intracranial volume

**Fig. 6:**
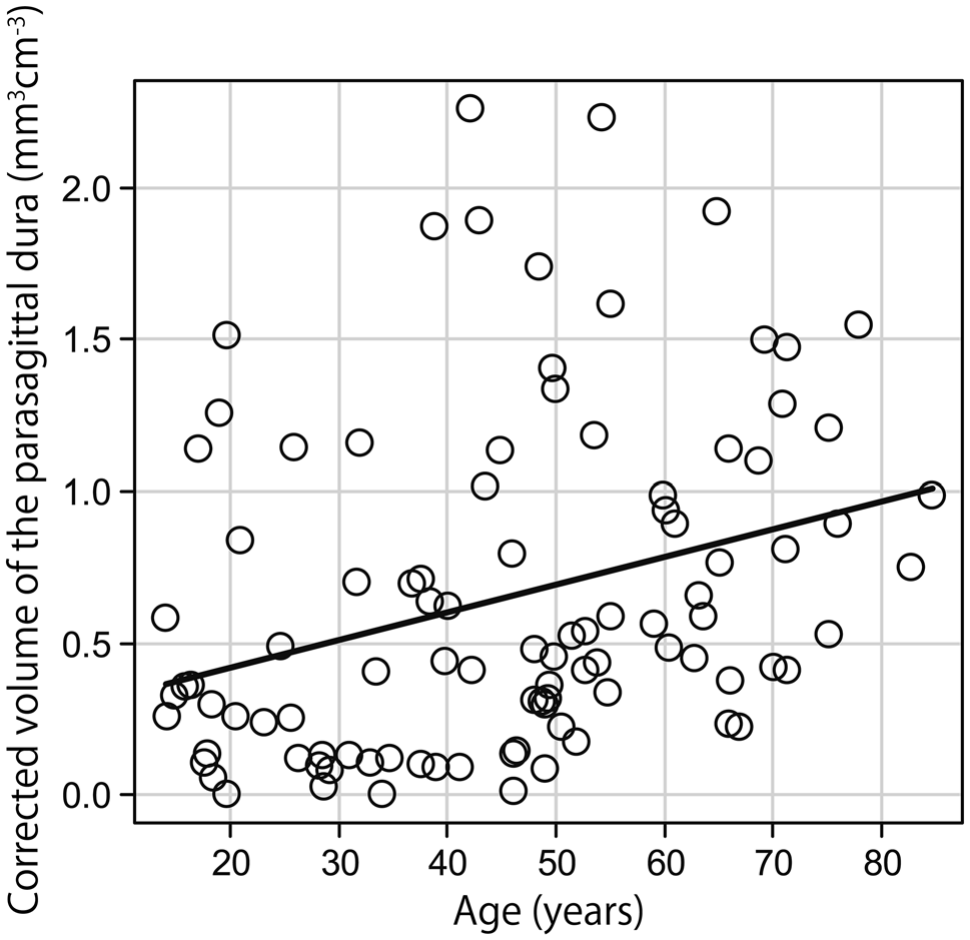
A significant weak positive correlation (*r* = 0.385, *p* < 0.001) between age and the volume of the parasagittal dura corrected for intracranial volume

**Fig. 7:**
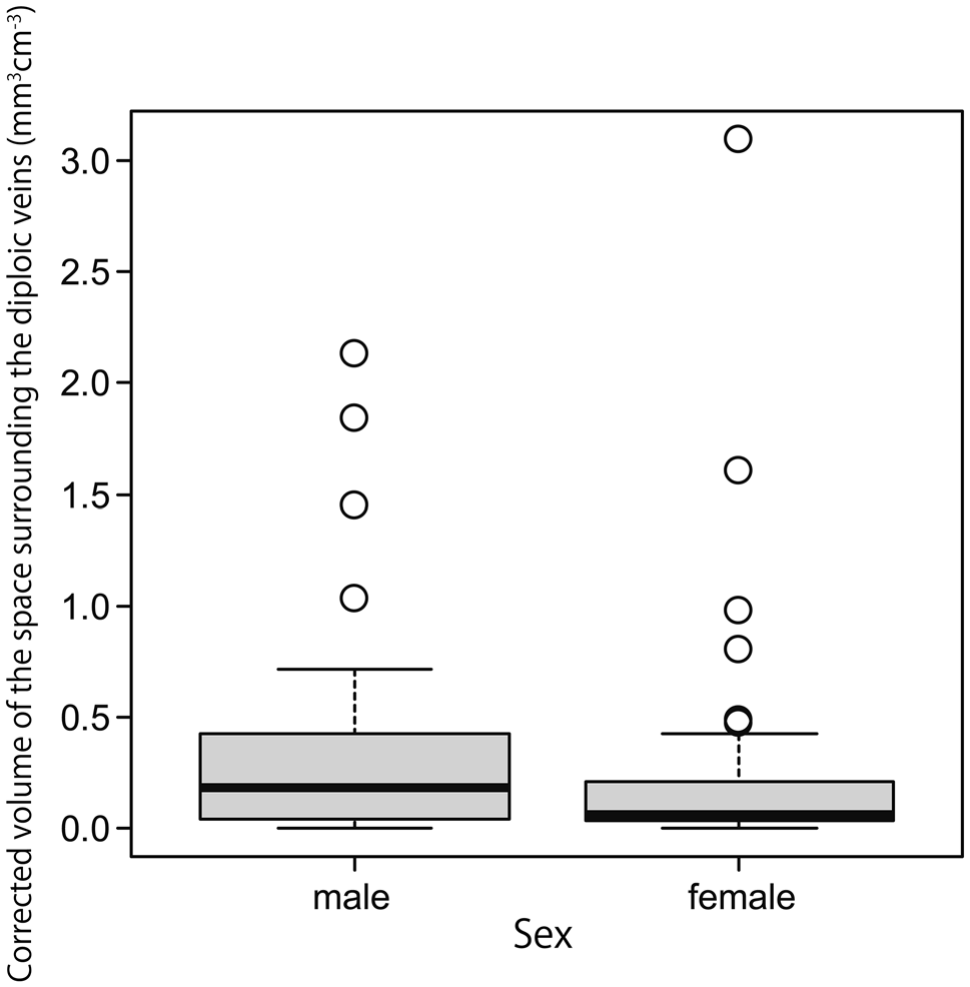
Relationship between sex and the volume of the space surrounding the diploic veins corrected for intracranial volume, showing a significantly greater volume in men than in women (*p* = 0.047)

**Fig. 8:**
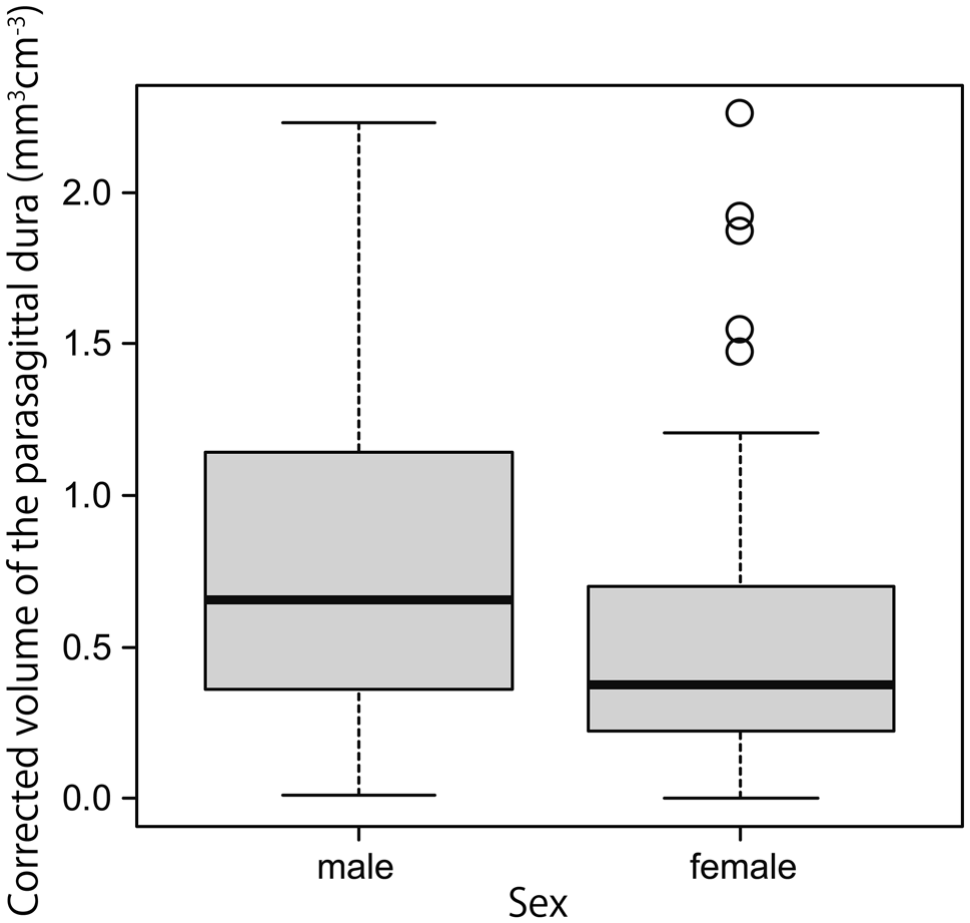
Relationship between sex and the volume of the parasagittal dura corrected for intracranial volume, showing a significantly greater volume in men than in women (*p* = 0.014)

The Kolmogorov–Smirnov test revealed that the participants’ ages were normally distributed. However, the volume of the space surrounding the diploic veins and the volume of the PSD were not normally distributed when both the corrected and uncorrected volumes were evaluated. After correcting for intracranial volume, the median volume of the space surrounding the diploic veins and the volume of the PSD were 0.102 (interquartile range, 0.037 to 0.324) mm^3^ cm^−3^ and 0.482 (interquartile range, 0.239 to 0.987) mm^3^ cm^−3^, respectively. Uncorrected for intracranial volume, the median volume of the space surrounding the diploic veins and the volume of the PSD were 158.057 (interquartile range, 55.273 to 511.280) mm^3^ and 657.593 (interquartile range, 356.775 to 1435.218) mm^3^, respectively. The correlations of age with the volume of the space surrounding the diploic veins and the volume of the PSD corrected for intracranial volume are shown in Figs. 5 and 6, respectively. The relationships of sex with the volume of the space surrounding the diploic veins and the volume of the PSD corrected for intracranial volume are shown in Figs. 7 and 8, respectively.

There was a weak but significant negative correlation between age and the volume of the space surrounding the diploic veins corrected for intracranial volume (*r* = −0.330, *p* < 0.001) and a significant weak positive correlation between age and the volume of the PSD corrected for intracranial volume (*r* = 0.385, *p* < 0.001). A similar correlation was observed when both volumes were not corrected for intracranial volume (volume of the space surrounding the diploic veins: *r* = −0.328, *p* < 0.001; PSD volume: *r* = 0.362, *p* < 0.001).

No correlation was found between the volume of the space surrounding the diploic veins and the volume of the PSD.

The volume of the space surrounding the diploic veins corrected for intracranial volume tended to be significantly greater in men than in women (*p* = 0.047). The volume of the PSD corrected for intracranial volume also tended to be significantly greater in men than in women (*p* = 0.014). A similar trend was observed when neither volume was corrected for intracranial volume (volume of the space surrounding the diploic veins: *p* = 0.017; PSD volume: *p* = 0.003).

### Inter-rater reliability

Neither the volume of the space surrounding the diploic veins nor the volume of the PSD in 10 randomly selected participants, as measured by two radiologists, was normally distributed. Therefore, the logarithm of these volumes (Ln(*x* + 1)) was calculated. The Kolmogorov–Smirnov test was performed on the calculated logarithms, which were all normally distributed. Using these logarithms, the ICC (2, 1) between the measurements of the ROIs made by the two radiologists was calculated to be 0.971 [95% confidence interval (CI), 0.886–0.993] for the volume of the space surrounding the diploic veins and 0.997 (95% CI, 0.985–0.999) for the volume of the PSD.

## Discussion

In this study, we visualized and measured the distribution of the GBCA into the space surrounding the diploic veins on MSDE T1-weighted images. The volume of the space surrounding the diploic veins was negatively correlated with age and was greatly developed in several younger participants. These findings contrast with the positive correlation found in the present study and previous reports [11–13] between age and the volume of the PSD, which is thought to be involved in the excretion of intracranial waste products along with meningeal lymphatics. Recently, skull–meninges connections in the vicinity of the diploic veins have been reported in humans, and at the same time, only the skull, among all the bones in the body, has a characteristic molecular profile associated with migration and inflammation in the bone marrow system [1]. Molecular translocation from the cerebrospinal fluid to the diploe has also been observed, suggesting that the cranial diploe is involved in cerebral immune surveillance [2]. Furthermore, aquaporin 4 is expressed only in the cranial diploe among all the bones in the body [14]. In the diploe, the space surrounding the diploic veins, which we suggest may be involved in cerebral immunity and cerebrospinal fluid/intracranial waste excretion, may change in volume depending on the situation (e.g., increasing in size with increased cerebrospinal fluid excretion). Therefore, we consider that it is important to objectively measure the volume of the space surrounding the diploic veins.

The diploic veins running within the diploe of the skull were discovered in the nineteenth century by the French anatomist and surgeon Guillaume Dupuytren. The diploic veins are continuous with the SSS [3]. The diploic veins function as collateral pathways when the SSS is occluded due to meningioma or other causes [4, 5]. The diploic veins are continuous with the arachnoid protrusion into the skull, suggesting their involvement in cerebrospinal fluid excretion and intracranial waste excretion [6]. In a study on formalin-fixed cadaveric heads, diploic veins were most abundant in the parietal bone [15]. Several reports have evaluated the distribution of diploic veins on imaging, with some reporting them as more common in the occipital region [16] and others finding them to be more common in the frontal and temporal regions [17] or in the frontal and parietal bones [18]. A report that categorized the diploic veins into four pathways revealed the most common pathway to be the pteriofrontparietal pathway, which was found bilaterally in 98% of patients [19]. This is considered to be included in the range of measurements of the space surrounding the diploic veins in the present study. Although the distribution of diploic veins has been reported to be poorly correlated with age, sex, and cranial volume [18], we found a significant weak negative correlation between the volume of the space surrounding the diploic veins and age, with and without correction for intracranial volume. The space surrounding the diploic veins may play a greater role in facilitating the excretion of cerebrospinal fluid and intracranial waste products in younger individuals. In addition, the volume of the space surrounding the diploic veins tended to be significantly greater in men than in women, regardless of whether it was corrected for intracranial volume. This finding suggests that the pathways and efficiency of intracranial waste excretion may differ between men and women.

Recent studies on the mechanism of brain waste removal have reported the presence of a lymphatic system in the brain [20, 21]. Meningeal lymphatics were identified along the SSS and visualized in human participants by contrast-enhanced MRI [22]. However, later studies suggested that this visualized PSD may not be a true meningeal lymphatic vessel but a bridging space that allows for cerebrospinal fluid-mediated molecular exchange between the meningeal lymphatics and brain tissue [23, 24]. It has been suggested that PSD is involved in the excretion of interstitial fluid and waste products as a downstream pathway of the glymphatic system [25, 26], and its possible association with brain immunity has also been reported [11]. Recently, a significant correlation between the PSD volume and β-amyloid accumulation has been reported [27], increasing the importance of accurate volume measurements of the PSD. In terms of imaging of the PSD, contrast-enhanced fluid-attenuated inversion recovery (FLAIR) and T1-weighted images [22], 3D T1-weighted black-blood MRI [11, 12], and simple T2-weighted images [13] have been used. In this study, the volume of the PSD was measured using contrast-enhanced T1-weighted images with three-axis MSDE performed 4 h after intravenous GBCA injection on 3-T MRI. Our findings regarding the correlation between the PSD volume and age and greater PSD volume in men than in women were consistent with those in previous reports [11–13]. The PSD showed an inverse correlation with the space surrounding the diploic veins according to age, suggesting that the PSD may have a complementary function to the space surrounding the diploic veins, although no direct negative correlation was observed. In addition, the PSD volume differed significantly between men and women, regardless of whether it was corrected for intracranial volume, suggesting that the distribution of intracranial waste excretion mechanisms or cerebral immune mechanisms may differ between men and women, and further study is warranted.

In this study, various attempts were made to avoid complications and errors and to improve reproducibility when measuring the volume of the space surrounding the diploic veins and the volume of the PSD on MSDE T1-weighted images. We measured only the slices above the superior border of the lateral ventricles, similar to how we previously measured the extent of contrast leakage into the subarachnoid space [28], to make comparisons based on a standardized area where volume measurements were taken. Limiting the area also reduced the measurement effort and consequently reduced the error. When measuring the volume of the space surrounding the diploic veins, only high-signal regions with a diameter of ≥2 mm were measured. When measuring the volume of the PSD, only the high-signal areas within a 1 cm area from the outer edge of the SSS were measured. The threshold-based methods used for these measurements were described previously [12]. Thus, the inter-rater reliability of the volume measurements was sufficient owing to several factors, including the abovementioned attempts.

This study has some limitations. First, selection bias may have existed because all the included participants were patients with suspected endolymphatic hydrops, and none were completely healthy. Second, there is no standardized way to spend 4 h after GBCA injection. The effects of movement, eating, and drinking may be confounding variables that require further investigation. Third, the lack of histologic evidence regarding the space surrounding the diploic veins prohibits definitive determination of its nature, raising the possibility of alternative structures, such as bone marrow. However, given that the high-signal regions exceeding the threshold on the MSDE T1-weighted images were predominantly observed surrounding the diploic vein (Fig. 2), and reticular high-signal regions exceeding the threshold (Fig. 3) were only sporadically detected in certain participants, we consider it unlikely that they represent bone marrow. Fourth, volume measurements were obtained only for the slices above the superior margin of the lateral ventricles, and not all of the cranium could be evaluated. Fifth, the space surrounding the diploic veins, which were <2 mm in diameter or indistinguishable from the reticular high-signal regions exceeding the threshold, was not measured and may be underestimated.

In conclusion, the distribution of the GBCA into the space surrounding the diploic veins was measured on MSDE T1-weighted images, and we demonstrated that this volume was negatively correlated with age. Our findings suggest that the space surrounding the diploic veins is greater at younger ages and may play a role in the excretion of cerebrospinal fluid and waste products and in immune responses during youth.

## Data Availability

The participants in this study did not provide written consent for their data to be shared publicly; due to the sensitive nature of the research, supporting data are not available.
